# A Self-Organizing Spatial Clustering Approach to Support Large-Scale Network RTK Systems

**DOI:** 10.3390/s18061855

**Published:** 2018-06-06

**Authors:** Lili Shen, Jiming Guo, Lei Wang

**Affiliations:** 1 School of Geodesy and Geomatics, Wuhan University, Wuhan 430079, China; llshen@sgg.whu.edu.cn; 2 State Key Laboratory of Information Engineering in Surveying, Mapping and Remote Sensing, Wuhan University, Wuhan 430079, China

**Keywords:** network RTK, spatial clustering, self-organizing spatial clustering (SOSC), precise positioning

## Abstract

The network real-time kinematic (RTK) technique can provide centimeter-level real time positioning solutions and play a key role in geo-spatial infrastructure. With ever-increasing popularity, network RTK systems will face issues in the support of large numbers of concurrent users. In the past, high-precision positioning services were oriented towards professionals and only supported a few concurrent users. Currently, precise positioning provides a spatial foundation for artificial intelligence (AI), and countless smart devices (autonomous cars, unmanned aerial-vehicles (UAVs), robotic equipment, etc.) require precise positioning services. Therefore, the development of approaches to support large-scale network RTK systems is urgent. In this study, we proposed a self-organizing spatial clustering (SOSC) approach which automatically clusters online users to reduce the computational load on the network RTK system server side. The experimental results indicate that both the SOSC algorithm and the grid algorithm can reduce the computational load efficiently, while the SOSC algorithm gives a more elastic and adaptive clustering solution with different datasets. The SOSC algorithm determines the cluster number and the mean distance to cluster center (MDTCC) according to the data set, while the grid approaches are all predefined. The side-effects of clustering algorithms on the user side are analyzed with real global navigation satellite system (GNSS) data sets. The experimental results indicate that 10 km can be safely used as the cluster radius threshold for the SOSC algorithm without significantly reducing the positioning precision and reliability on the user side.

## 1. Introduction

The network real-time kinematic (RTK) technique is one of the most popular global navigation satellite system (GNSS)-based precise positioning techniques, and provides instantaneous centimeter-level accuracy positioning solutions within its service area. The network RTK has a larger coverage area than the single reference station RTK system, and its coverage area can be further extended by installing more reference stations [1]. Therefore, it can be considered as a national or provincial geo-spatial infrastructure to support future precise positioning applications [2]. The network RTK system collects the observations from the global navigation satellite systems (GNSSs) and then establishes regional ranging error models and computes the corrections for the users. Users take advantage of the corrections to mitigate the error sources and improve their positioning performance. Therefore, network RTK systems are also considered as ground-based augmentation systems (GBASs). There are many network RTK implementation methods, including the virtual reference station (VRS) [3], the Master Auxiliary Concept (MAC) [4], pseudo-reference stations (PRSs), individualized master auxiliary corrections (i-MAX), and the flächen-korrektur parameter (FKP) [5]. These methods are more or less similar in terms of theoretical aspects, and the two most representative network RTK implementations are the virtual reference station (VRS) and regional correction (FKP). The two implementations achieve similar positioning performance, but the corrections are expressed in different forms. The FKP approach broadcasts the region-specified corrections and the users need to interpolate the corrections by themselves. The VRS approach provides an individualized correction service for each customer. Customers report their approximate position first and then the server generates observations of a virtual reference station near the user’s location. In this way, the user can adopt the VRS correction as with the conventional RTK without any modification of receiver firmware. The VRS avoids compatibility issues and modification of the user–receiver algorithm, and thus has become more popular than the FKP approach [6]. The VRS approach requires a two-way communication link between the server and the users. The two-way communication link also benefits online user management, for example in geo-fencing and charging. The challenge of VRS approach is that the computational burden of the server may dramatically increase as the concurrent user number increases.

In the past, the network RTK technique was mostly oriented towards professional applications such as surveying, mapping, and deformation monitoring, etc., since the mass market had little high-precision positioning requirement. Most current provincial network RTK systems only support up to a few hundred concurrent online users. In recent years, a need for precise positioning has arisen in the massive market, driven by developments in artificial intelligence, unmanned aerial vehicles (UAVs), and robotics [7]. Many autonomous vehicles [8] as well as robotics equipment and UAVs require precise positioning, and the network RTK technique has become the first choice [9,10,11]. In the future, millions of smart vehicles and devices will need to access the precise positioning service from network RTK systems. An issue will thus be how to support massive numbers of concurrent users with current network RTK systems. Therefore, how to reduce the computational burden on the server side becomes a key issue for supporting the massive network system. The computational burden can be mitigated by allocating more computing resources, such as grid computing or cloud computing [12]. The limitation of this method is increasing investment and system complexity. Instead, computation burden mitigation by improving the algorithm is more cost-effective. A predefined grid approach has been proposed as an improvement on the algorithm, which clustering the users with a predefined cluster center [13]. This is a patented technology which generates a set of VRSs at predefined grid locations, and users form double-differenced observations between the real observations and the nearest VRS. This method partially solved the problem since the number of VRSs can be controlled on the server side, but it does not consider the user’s location, so it is complex to reasonably determine the site of the VRS. Recent research indicates that the largest Chinese network RTK system operator, which maintains over 1300 GNSS permanent stations, has adopted the predefined grid approach for VRS generation [14]. In this study, we proposed a solution known as the self-organizing spatial clustering (SOSC) approach for computation burden mitigation on the network RTK system server side. The proposed approach can determine the optimal VRS number and location according to the spatial distribution of user locations, without supervision. Moreover, the impact of spatial clustering on positioning performance on the user side is analyzed with real GNSS data sets, and the safe cluster radius is determined accordingly.

## 2. The Procedure of Network RTK-Based Precise Positioning

**Figure 1:**
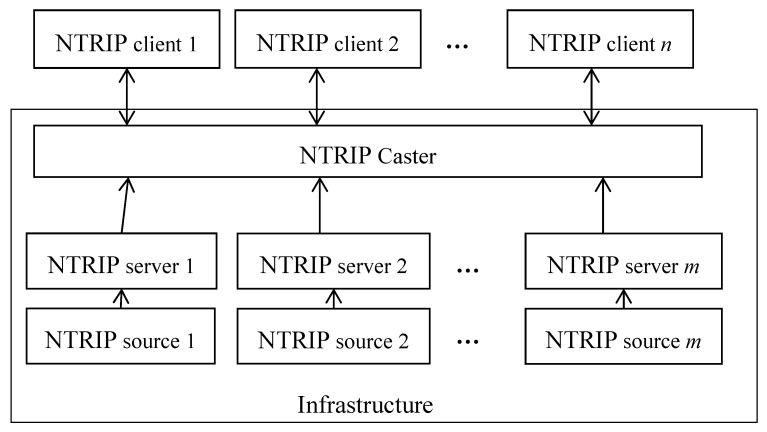
The logical structure of the network real-time kinematic (RTK) system. NTRIP: Networked Transport of RTCM via Internet Protocol.

The conventional RTK system is designed for radio transmission. Therefore, corresponding data transmission protocols, such as the Radio Technical Commission for Maritime services (RTCM), compact measurement record (CMR) [15] etc., are not compatible with the network environment. The Networked Transport of RTCM via Internet Protocol (NTRIP) is designed for wrapping existing protocols to fit the network transmission environment [16]. The protocol divides the network RTK systems into four parts: the NTRIP source, the NTRIP server, the NTRIP caster, and the NTRIP client [17]. The logical structure of a network RTK system can be illustrated by Figure 1. The reference stations continuously track GNSS signals and serve as the NTRIP sources. A network RTK system involves many reference stations with an inter-station distance of 40–70 km [18]. The NTRIP servers convert the observation data from the reference station into real-time streams and deliver the streams to the NTRIP caster. The NTRIP caster is the data center of the network RTK system, which receives the real-time data streams from the NTRIP servers and dispatches correction data streams to the NTRIP clients. The NTRIP caster is also responsible for the VRS generation from the observation data streams. The NTRIP clients are users of the network RTK system, and receive correction data streams to compute their own high-precision positions. The NTRIP clients employ duplex data link to the NTRIP caster for VRS-based network RTK systems. They report their location to the NTRIP caster first and then receive the VRS correction data. The NTRIP sources, the NTRIP servers, and the NTRIP caster form the infrastructure of the network RTK system, which supports varying numbers of NTRIP clients. The number of supported concurrent online NTRIP clients is known as the capacity of the network RTK system. With the same network RTK infrastructure, higher capacity means greater benefit.

### 2.1. The Procedure of VRS Generation

**Figure 2:**
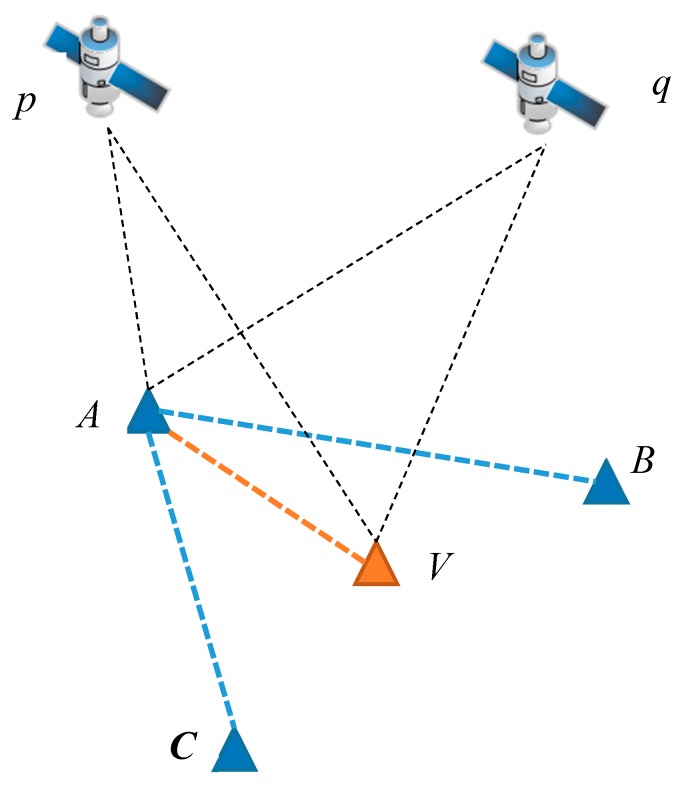
Demonstration of virtual reference station (VRS) generation.

The generation of VRSs is the key to the network RTK systems. The simplest network RTK system includes three reference stations and one VRS, as demonstrated in Figure 2. The GNSS observation equation [19] is as follows:

(1) $$\begin{array}{l} P_{i}=\rho +\delta_{orb}+\delta t^{s}+\delta t_{r}+T+I_{i}+B_{r,i}+B^{s,i}+MP_{i}+\epsilon \\ L_{i}=\rho +\delta_{orb}+\delta t^{s}+\delta t_{r}+T-I_{i}+\lambda_{i}N_{i}+b_{r,i}+b^{s,i}+MP_{i}+\epsilon \end{array}$$

where $P_{i},L_{i}$ are the pseudorange and carrier phase measurement, respectively, of the ith frequency, expressed in meters. ρ, $\delta_{o\mathrm{rb}}$ are the geometrical distance and GNSS orbit error, respectively. For a baseline case of less than 100 km, the orbit error is assumed to be fully eliminated by the inter-station difference operation. $\delta t^{s}$ and $\delta t_{r}$ are the satellite clock bias and receiver clock bias, respectively. T and $I_{i}$ are the propagation path-specified error, known as the tropospheric delay and ionospheric delay, respectively. $B_{r,i}$ and $B^{s,i}$ are the receiver-specified and satellite-specified code bias. $b_{r,i}$ and $b^{s,i}$ are the initial phase fractional biases. The presence of $b_{r,i}$ and $b^{s,i}$ contaminates the integer nature of ambiguity parameter in zero difference mode. $MP_{i}$ and ϵ are the multipath error and measurement noise, respectively.

The basic idea of the VRS is the generation of a set of observations virtually observed at point *V* with real observations from points *A*, *B*, and *C*. The VRS observations can be generated by adding corrections to the real measurements. The virtual observations at station *V* can be expressed by:

(2) $$\begin{array}{l} P_{i,V}=P_{i,A}+\Delta P_{i,AV}\\ L_{i,V}=L_{i,A}+\Delta L_{i,AV} \end{array}$$

where the subscripts V and A indicate the observation station. Δ is the inter-station difference operator. The inter-station difference eliminates the satellite-specified error sources. In relative positioning mode, the receiver-specified errors are nuisance biases which are often handled by inter-satellite difference operation. Since there is no physical receiver clock, we simply assume the receiver-specified bias at the virtual station is exactly the same as for the real measurement. This assumption does not affect the positioning results since all the receiver-specified biases will be eliminated in relative positioning procedure at the user side.

Then, the inter-station difference observations between the station *A* and station *V* only contain the geometry difference and the propagation-specified error sources, which can be expressed as [20,21]:

(3) $$\begin{array}{l} \Delta P_{i,AV}=\Delta \rho_{AV}+\Delta T_{AV}+\Delta I_{i,AV}\\ \Delta L_{i,AV}=\Delta \rho +\Delta T_{AV}-\Delta I_{i,AV}+\lambda_{i}\Delta N_{i,AV} \end{array}$$

The equation indicates the remaining terms in the inter-station differenced observations are the geometrical distance, propagation path specified errors and the ambiguity parameters.

The remaining problem is how to compute the single-difference measurements $\Delta P_{i,AV}$ and $\Delta L_{i,AV}$. The VRS is determined according to the report from the NTRIP clients, so its coordinates are precisely known. The coordinates of the reference stations *A*, *B*, and *C* can be precisely predetermined, since they are considered still during the network RTK system operation period. Then the geometrical distance displacement term $\Delta \rho_{AV}$ can be precisely computed. The atmospheric delay at station V can be interpolated from the baselines *AB* and *AC*. The baseline *AB* and *AC* are estimated in double-difference mode, which provides the double-differenced atmospheric delay $\nabla \Delta T_{AB}^{p,q}$, $\nabla \Delta T_{AC}^{p,q}$, $\nabla \Delta I_{i,AB}^{p,q}$, and $\nabla \Delta I_{i,AC}^{p,q}$, where ∇Δ is the double-difference operator. The superscript indicates inter-satellite difference between satellites *p* and *q*, while the subscript indicates the inter-station difference between stations *A*, *B* or *A*, *C*.

It is noted that the network processing can only provide a double-differenced atmospheric delay, while VRS requires a single-difference atmospheric delay. Assuming the satellite *p* is the reference satellite, the required single-difference atmospheric delay for satellite *q* can be expressed as:

(4) $$\begin{array}{l} \Delta T_{AV}^{q}=\Delta T_{AV}^{p}+\nabla \Delta T_{AV}^{pq}\\ \Delta I_{i,AV}^{q}=\Delta I_{i,AV}^{p}+\nabla \Delta I_{i,AV}^{pq} \end{array}$$

The double-differenced terms $\nabla \Delta T_{AV}^{pq}$ and $\nabla \Delta I_{i,AV}^{pq}$ can be interpolated from the network processing with the linear interpolation method (LIM), the low-order surface model (LSM), the linear combination model (LCM), the distance-based linear interpolation method (DIM), the least-squares collocation (LSC) method [22], or the Kriging interpolation algorithms [23]. Although the bias terms $\Delta T_{AV}^{p}$ and $\Delta I_{i,AV}^{p}$ are unknown, they are the same for all visible satellites, so they can be automatically assimilated into the receiver clock term or eliminated by inter-satellite difference operation. The transformation of the double-difference operator into a single-difference operator is also known as the datum transformation of ‘S-basis transformation’, which is also widely used in satellite clock determination, precise point positioning-RTK (PPP-RTK) [24], network data processing, and rank defect network adjustment, etc. [25]. As the initial phase term is eliminated, the integer nature of ambiguity parameter is preserved. Although the reconstructed VRS observations are basis-shifted, the basis transformation has no impact on relative positioning on the user side.

### 2.2. The Bottleneck of Large-Scale Network RTK Systems

As discussed in previous section, the network RTK systems perform atmospheric delay interpolation and geometrical correction, and generate VRS observations for every online user in real-time mode. The computational burden will dramatically increase as the concurrent online user number increases. Moreover, the corrections of the network RTK systems have to be generated in real time, since the latency may degrade positioning performance on the user side. If the corrections from VRS are not synchronized with the observations at the user end, the propagation path-dependent error may increase since both ionospheric delay and tropospheric delay change over time. The remaining error will be assimilated by the coordinate parameters and thus degrade the positioning precision on the user side. The current network scheme is only suitable for supporting professional applications with limited concurrent users. When the network RTK technique is extended into the mass market, the smart vehicles, the robotics and the UAVs all become network RTK users. As a spatial infrastructure, the network RTK is responsible for providing a quality-ensuring differential correction service to support all clients. However, currently a user-oriented VRS service is not possible for supporting large numbers of concurrent users.

One possible solution is employing more power computing resources such as grid computing or cloud computing, although this increases the system complexity and cost. A more cost-effective solution is the reduction of the computational burden by improving the algorithms.

**Figure 3:**
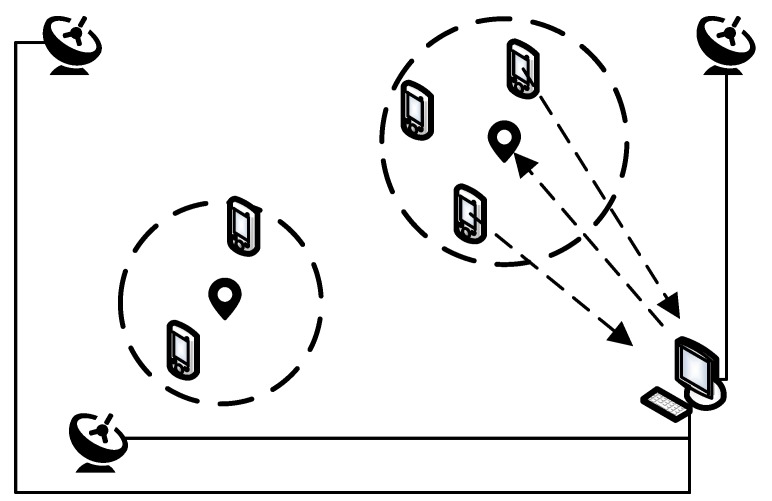
Illustration of the spatial clustering approach for the large-scale network RTK system.

As stated, the major challenge of VRS generation is handling the propagation path-dependent biases. These biases are strongly correlated when two stations are close, and the spatial correlation weakens as the distance increases. Therefore, it is reasonable to share the same VRS information if two clients sit close to each other. In this way, we propose a spatial clustering algorithm, which clusters the clients into groups first and each group shares the same VRS information. After clustering, the unlimited client number can be mapped into limited service area. Hence, the maximum VRS number can be guaranteed with a limited network RTK service area. The concept of the spatial clustering approach can be illustrated by Figure 3. In the figure, the NTRIP caster generates five virtual stations in current network RTK systems since there are five online users. Some of the clients stand close to each other since they are working on the same project or within the same business district. Considering the spatial correlation, two independent VRS are good enough to serve all five clients. With a proper cluster radius, it is possible to achieve comparable positioning performance with only two VRSs as compared to the five-VRS solution, so the computational burden is reduced.

### 2.3. Self-Organizing Spatial Clustering Algorithm

The clustering methods are highly non-unique, and can be generally classified as distance- or similarity-based clustering, hierarchical clustering, density-based clustering, graph theory-based theory, neural network-based clustering, kernel-based clustering, and the mixed clustering method [26,27]. With regard to the large-scale network RTK systems, the predefined grid algorithm has been proposed to mitigate the computational burden in network RTK servers. The predefined grid algorithm generates VRSs at predetermined grid points and delivers the best VRS for each NTRIP client. The method considers the spatial correlation between the NTRIP clients but the location of the VRS may not be always optimal since the spatial distribution of clients is not considered. Different clustering methods have been designed for solving different problems. The density-based clustering algorithm is the best choice for the network RTK optimization problem. There are a number of density-based clustering algorithms in the machine learning field, such as the density-based spatial clustering of applications with noise (DBSCAN) [28], ordering points to identify the clustering structure (OPTICS) [29], and density-based clustering (DENCLUE) [30]. However, none of the existing algorithms can fit the requirement of this particular problem. The constraints of the clustering algorithms in network RTK systems can be summarized as follows:

(1) The radius of cluster constraint: Since the spatial correlation decreases as the baseline length increases, the radius of the cluster has to be controlled to ensure the positioning performance.
(2) The real-time constraint: the network RTK system is a real-time system, so the clustering algorithm has to be computationally efficient.
(3) All users should be properly clustered and no negligible user is allowed.

The famous DBSCAN algorithm and OPTICS algorithm may produce isolated points, so they cannot be directly used in this application. The DENCLUE algorithm is robust to noise [31], but it requires iteration, so it is considered not efficient enough. In order to meet the requirements, a new density-based clustering algorithm is proposed and named the self-organizing spatial cluster (SOSC) algorithm. The procedure of the SOSC algorithm (Algorithm 1) is briefly summarized as follows:

**Algorithm 1.** SOSC.  Given alternative set $U=\;\{x_{1},x_{2},\cdots ,x_{n}\}$, where $x_{i}$ is the position of ith NTRIP client and total online client number is n:
(1)Create an empty cluster set $C_{j}=\emptyset$, put one arbitrary element $x_{i}\in U$ into $C_{j}$, and remove it from U; let $p_{j}=x_{i}$, where $p_{j}$ is the initial center of the cluster $C_{j}$.(2)Get one arbitrary element $x_{i}\in U$; go to step 3.(3)Check if $\|x_{i}-p_{j}\|\leq \mu$, and then add $x_{i}$ into $C_{j}$ and remove it from U; update cluster center $p_{j}$, and go to step 4. The cluster center updating algorithm can be described as $p_{j}=\frac{p_{j}k+x_{i}}{k+1}$, where k is the number of points in cluster $C_{j}$. μ is the radius threshold of the cluster.If $\|x_{i}-p_{j}\|>\mu$, go to Step 2.(4)Check if U=∅, then exit; otherwise, go to step 5.(5)Check if $x_{i}$ is the last element in U, then go to step 1, otherwise go to step 2

**Figure 4:**
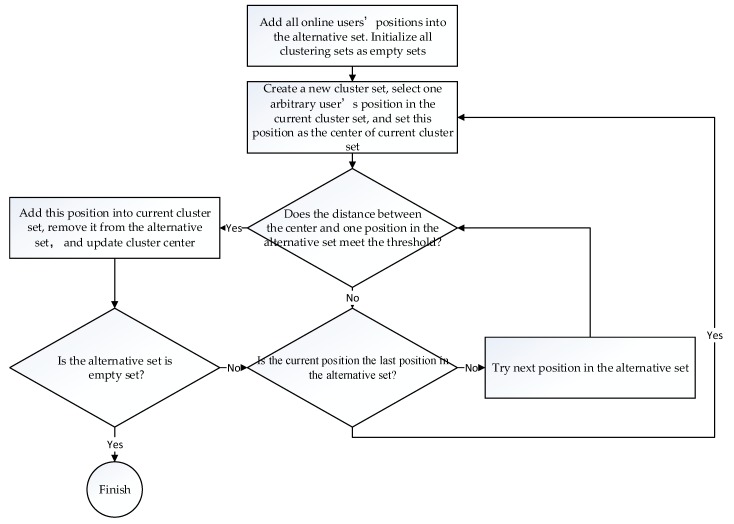
The flowchart of the self-organizing spatial clustering algorithm.

The benefits of the SOSC algorithm are three-fold. Firstly, the SOSC algorithm is an unsupervised clustering algorithm. It only requires one parameter—the radius threshold. The remaining work can be automatically finished with the algorithm. Secondly, the algorithm is computationally efficient, since the complexity of SOSC algorithm is O(n^2^). Thirdly, the algorithm can determine the center of clusters and the cluster number according to the spatial distribution of clients. The flowchart of the SOSC algorithm is depicted in Figure 4.

### 2.4. Parallel Computing for Ultra-Large Network RTK Systems

The complexity of the spatial clustering algorithm is O(n^2), which is fairly efficient, but its time consumption still increases as the concurrent client number increases. This means that there is a breakdown client number, which leads to the SOSC algorithm becoming overdue with a given latency constraint. The network RTK system is a real-time system, so the latency is an important system measure. Therefore, the breakdown point defines the volume of the network RTK systems. In order to further extend the system volume, the advanced computing algorithm can be employed.

**Figure 5:**
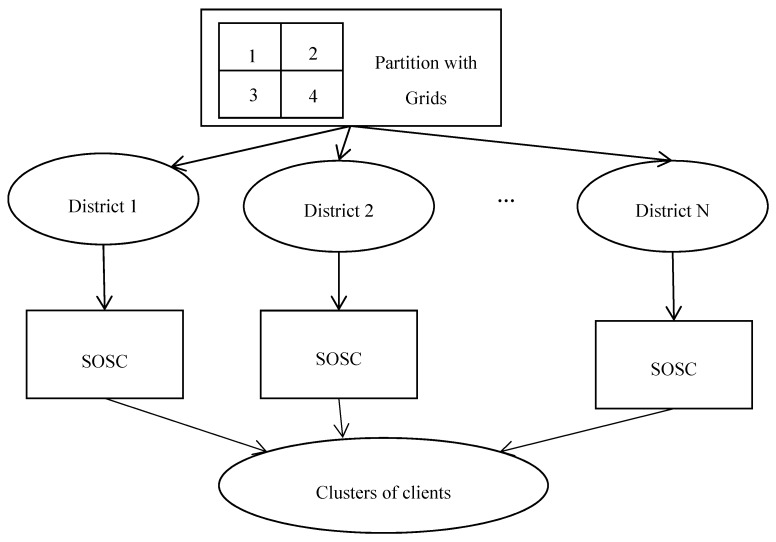
The scheme of parallel computing for ultra-large scale network RTK systems. SOSC: self-organizing spatial clustering.

The scheme of parallel computing can be depicted by Figure 5. The figure indicates that the parallel computation scheme of SOSC algorithm is somehow similar to Google’s map-reduce scheme [32]. The client clustering task is partitioned into many subsets according to latitude and longitude. Then, the SOSC algorithm acts as the reducer and performs clustering on each subset in parallel. After parallel computation, the clustering results are returned back to the main task and combined for the output. The challenge of parallel computing also comes from how to reasonably partition the task to balance the computational load.

According to Gustafson’s law, the potential speedup in latency of the whole task can be roughly estimated as in [33]:

(5) $$S_{l}(s)=1-p+sp$$

where $S_{l}$ is the speedup in latency of the whole task. s is the speedup in the latency of the execution of SOSC algorithm. p is the percentage of SOSC execution time in the whole clustering algorithm time.

## 3. Implementation Aspect of Large Scale Network RTK System

**Figure 6:**
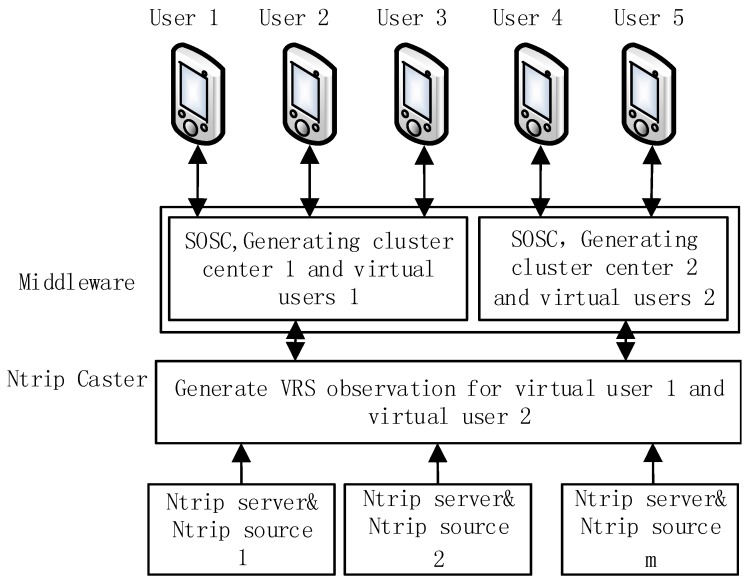
Integration of spatial clustering algorithm into the existing network RTK platform.

The proposed algorithm can be integrated into existing network RTK platform with the middleware technique. A demonstration of network RTK system with the middleware is presented in Figure 6. The middleware is deployed between the NTRIP clients and the NTRIP caster. The middleware accepts requests from the NTRIP clients and sends VRS data back to users. The middleware is also responsible for reorganizing the online NTRIP clients by the SOSC algorithm. The online clients are mapped into the clusters and the user location is replaced with the cluster center. After merging, virtual users are generated for each cluster and the information is sent to the NTRIP caster via the middleware. In this way, the existing NTRIP caster only computes the VRS for these virtual users. After the VRS is generated from the NTRIP server, the middleware maps the VRS information for the virtual users back to the physical users. The physical users in the same cluster receive the same VRS information. By deploying the middleware, the limitation of concurrent users are converted into the limitation of virtual users. The maps between the physical user and the virtual users are controlled by the middleware and the SOSC algorithm. This provides a potential capacity for supporting more concurrent online users.

## 4. Performance Evaluation

Although the SOSC algorithm is executed on the server side, it has potential impact on the positioning performance on the user side. Therefore, the performance of the spatial clustering approach should be evaluated from the server side and the user side, respectively. As for the server side, we focus on whether the clustering algorithm is efficient and whether the clustering results are reasonable, which can be examined with simulation data. At the user side, we determine a reasonable cluster radius for the SOSC algorithm with a real data test to avoid a potential negative impact on user positioning.

### 4.1. Performance Evaluation of the Server Side

On the server side, we focus on analyzing the benefit of the SOSC algorithm compared to existing algorithms. The benefit can be measured from two aspects: the computational efficiency and the reasonability of clustering. Since the network RTK system is a real-time system, it is unacceptable for incorporating algorithms with unpredictable latency. The most time-consuming task in the middleware is the SOSC algorithm, so computational efficiency affects bottlenecks in the real-time system. Another performance measure is the reasonability of clustering algorithm, which is measured by the cluster number and the mean distance to cluster center (MDTCC). The cluster number reflects whether the algorithm can efficiently group all clients within certain area into the same group. Improper cluster algorithm may split one cluster into many parts and thus produces a larger cluster number. The MDTCC is defined as:

(6) $$\mathrm{MDTCC}=\frac{1}{n}\sum_{i=1}^{n}\sqrt{{\left\|c_{i}-p\right\|}^{2}}$$

where $c_{i}$ and p are the position vector of ith client and cluster center, respectively. n is the total client number in the cluster. The equation indicates that MDTCC achieves a minimum only when the cluster center located at the geometrical center. In this case, the cluster center is considered as optimally determined. However, due to computational load constraints and the mobility of clients, it is difficult to determine the geometrical center. The predefined grid approach has been proposed to solve the large-scale network RTK system problem, so this method is used as the reference. This method predefines a set of cluster centers and then the network RTK server matches each client to the best cluster center, then all the clients are automatically clustered. Since the number of cluster centers is predefined, the VRS computation time also becomes controllable.

In order to evaluate the performance of the SOSC algorithm, a simulation is performed. In this study, the service area is simulated as a 200 km × 200 km square field. The cluster radius threshold for the SOSC algorithm is set as 10 km. In order to make the SOSC algorithm and the grid algorithm comparable, they should have the same cluster radius. Since no gap is allowed in the service area, the inter-station distance for the grid algorithm is 10$\sqrt{2}$ km rather than 20 km. As a result, 225 cluster centers are generated in total for the grid algorithm in the given service area.

For the clustering algorithm, we are only concerned with the location property of the clients so the simulation achieves exactly the same results as in practice, but the spatial distribution of client may affect the performance of the clustering algorithms. It is impossible to examine all possible distributions, so two typical distribution scenarios are considered: (1) uniform distribution; and (2) clustered distribution. The uniform distribution is the simplest case, where all clients are evenly distributed over the service area. This distribution is mathematically perfect, but this rarely happens in the real word. The clustered distribution means that the online users are gathered at several ‘hotspot’, these places may be construction site or fieldwork. This scenario is closer to the real world than the uniform distribution since people always clustered by their jobs or hobbies.

**Figure 7:**
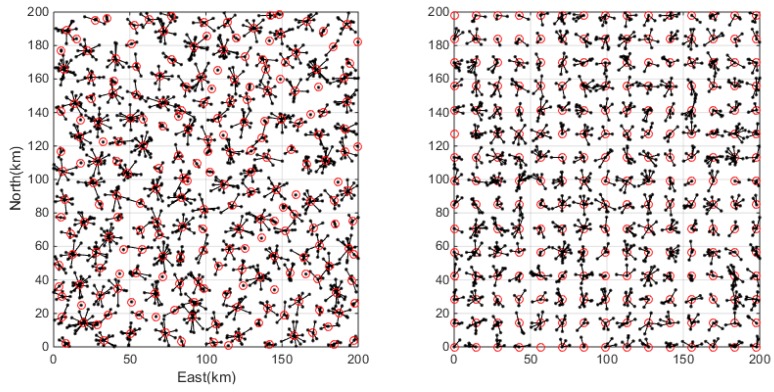
Clustering results with the SOSC (**left**) and grid (**right**) approaches for the uniform distribution case.

**Figure 8:**
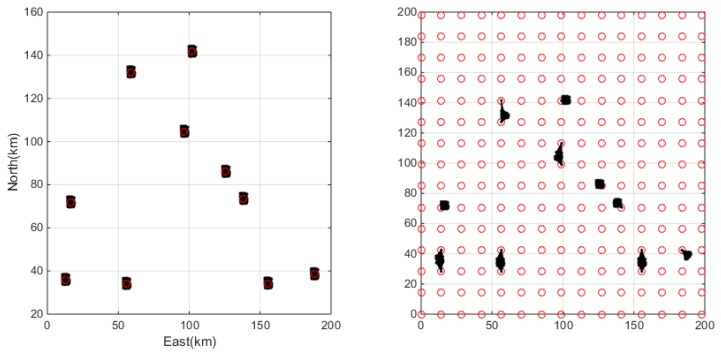
Clustering results with the SOSC (**left**) and grid (**right**) approaches for the clustered distribution case.

At first, the same data set involving 1500 user positions is used to test the clustering performance. The users are uniformly distributed over the service region and the clustering results by the SOSC algorithm and the grid algorithms are presented in Figure 7. In the figure, the black dots show the user positions and the red circles indicate the cluster centers. Clearly they are difficult to find, but it is noticeable that the SOSC algorithm always keeps the cluster centers in the center of clusters while the grid approach sometimes has all the points on the same side of the cluster center. The second experiment tests the performance of clustering algorithms under a clustered distribution. In this case, it assumes that all the clients are concentrated at several ‘hotspots’ and these hotspots may be their workplace, the central business district (CBD), or other public areas. This is more realistic case since it should not be expected that people follow a geographically uniform distribution. The clustering results under the clustered distribution are presented in Figure 8. The clustering results indicate that the SOSC algorithm always identifies a reasonable cluster center and cluster number, while the grid algorithm sometimes splits one cluster apart. This is because the grid approach is not sensitive to the distribution of clients.

**Figure 9:**
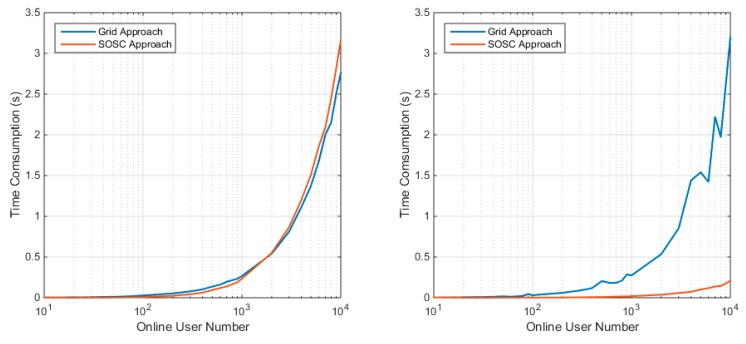
Time consumption of clustering with the grid approach and SOSC approach under uniform distribution (**left**) and clustered distribution (**right**).

The two figures give a first impression on how do these clustering algorithms works. The following section will compare their performance more comprehensively. As stated, the computational efficiency is one of the most important performance indicators of the clustering algorithm to evaluate the computational efficiency. The time consumption depends on the client number, so we tested client numbers ranging from 10 to 10,000 to determine their computational efficiency. The time consumption of SOSC approach against different online user number is evaluated and the results are presented in Figure 9. The figure gives time consumption of the two approaches under different user distributions. The left panel shows that the time consumption of both the grid algorithm and the SOSC algorithm dramatically increase as the online user number increases. For the uniformly distribution case, the two methods have similar time consumption. The SOSC approach slightly outperforms the grid approach when the online user number is smaller than 2000 and the grid approach outperforms the SOSC approach as the user number increases. Both methods can handle about 3500 concurrent users within 1 s. Considering the clustered distribution case, the SOSC approach significantly outperforms the grid approach. The computational efficiency of the grid algorithm does not change, while the SOSC algorithm improves dramatically. The simulation results indicate that the SOSC approach can properly handle over 10,000 online users within 0.2 s, while the grid algorithm takes more than 3 s. This is because the cluster number is greatly reduced by the SOSC algorithm, so the corresponding search procedure becomes more efficient, while the grid algorithm does not change.

The reasonability of clustering can be evaluated from two aspects: the cluster number and the mean distance to the cluster center (MDTCC).

**Figure 10:**
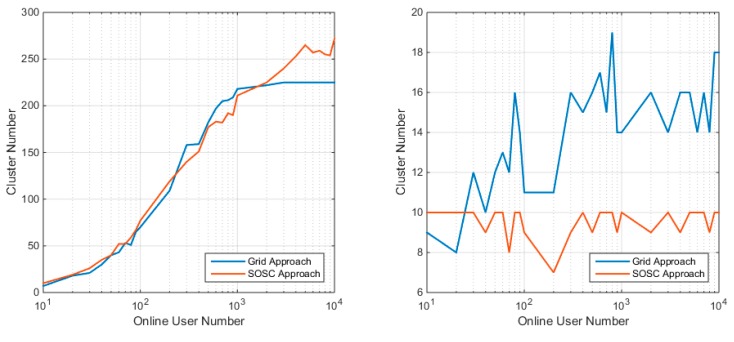
Cluster number of clustering with the grid approach and SOSC approach under uniform distribution (**left**) and clustered distribution (**right**).

With the given client position set and radius threshold, the smaller cluster number means the clustering algorithm is more efficient and consequently less computation required at the server side. Figure 10 gives the relationship between the online user number and the cluster number. The left panel shows that the SOSC approach and the grid approach produce similar cluster number under the uniform distribution. The difference is that the cluster number of the grid approach is no larger than 225, while SOSC approach has no theoretical upper bound. Fortunately, the cluster number of the SOSC approach does not go wild as the client number increases. This is because the clients are dense enough. The maximum cluster number of SOSC is about 270, which is slightly larger than in the grid approach. Considering the clustered distribution, the SOSC approach gives smaller cluster number. Actually, the SOSC approach gives its cluster number no larger than 10, which is the theoretical upper bound. In contrast, the grid approach is more likely to give the cluster number higher than 10 since the grid approach may split users from one cluster into multiple groups.

**Figure 11:**
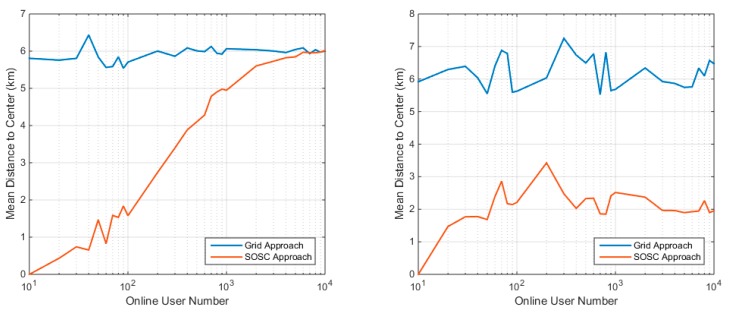
The mean distance to cluster center of different clustering approach under uniform distribution (**left**) and clustered distribution (**right**).

The mean distance to cluster center (MDTCC) is used to describe the optimality of the cluster center. With a given client position set, the clustering algorithm with minimum MDTCC should be the optimal clustering algorithm. However, due to the latency constraint, the SOSC is a sub-optimal clustering algorithm, which represents trade-off between the MDTCC indicator and the computational efficiency. Therefore, the SOSC algorithm cannot ensure production of the global smallest MDTCC. The MDTCCs of the SOSC approach and the grid approach are presented in Figure 11. The figure shows that the MDTCC of the grid approach is quite stable under different distributions and different client numbers, and lies between 6 km and 7 km. The MDTCC of SOSC approach is much better than the grid approach, although it is only a sub-optimal solution. The MDTCC of the SOSC approach increases as the client number increasing under the uniform distribution scenario. As the client becomes denser, the discrepancy of MDTCC between the SOSC approach and the grid approach disappears. The right panel indicates that the MDTCC of the SOSC approach is much better than the grid approach, which also indicates that the SOSC approach is more reasonable than the grid approach for the clustered distribution scenario.

### 4.2. Performance Evaluation of the User Side

The clustering-based approach can mitigate the computational load on the server side, but it also has a side effect on the user side. For the existing algorithms, the NTRIP server generates one VRS for each client, so the distance between the VRS and the clients can always be guaranteed. After clustering, the maximum distances between the VRS and the clients are constrained by the cluster radius threshold. A small cluster radius threshold means larger cluster number and heavier computational burden, while larger threshold means weaker spatial correlation between the client and VRS. Therefore, optimally determination of the cluster radius threshold is vital for the clustering-based approach. In this section, we attempt to address the side effects of longer distances between the VRS and the client on user side positioning, and find an optimal trade-off between the computational burden and positioning performance.

Considering the complexity of the positioning scenario, the real GNSS data set is employed in user positioning assessment. We collected our own observation data and VRS corrections were obtained from the commercial continuous operating reference stations (CORS) network. The target of this experiment is to determine the optimal cluster radius threshold of the SOSC algorithm. The test includes the static test and kinematic test. Both of them involve VRSs from difference places.

**Figure 12:**
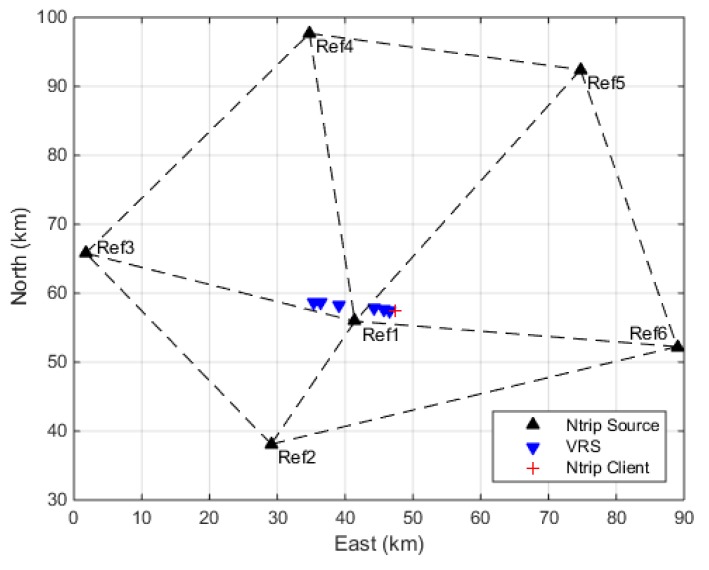
Distribution of the reference stations of the tested network RTK system.

For the static test, we collect real data from a commercial CORS network to verify the impact of distance between VRS and users on the positioning performance. The CORS network is demonstrated in Figure 12. The selected CORS network involves six reference stations with inter-station distance varying from 40 km to 60 km. We obtain the VRS corrections by reporting different locations to the network RTK system. The distance between VRS and the NTRIP client varies from 0.7 km to 12.1 km. On the user side, the GNSS antenna is mounted at a fixed monument with known coordinates. Trimble NetR9 receiver is adopted for the data collection. The data was collected from 22 to 24 January 2018, with session lengths of 24 h.

**Figure 13:**
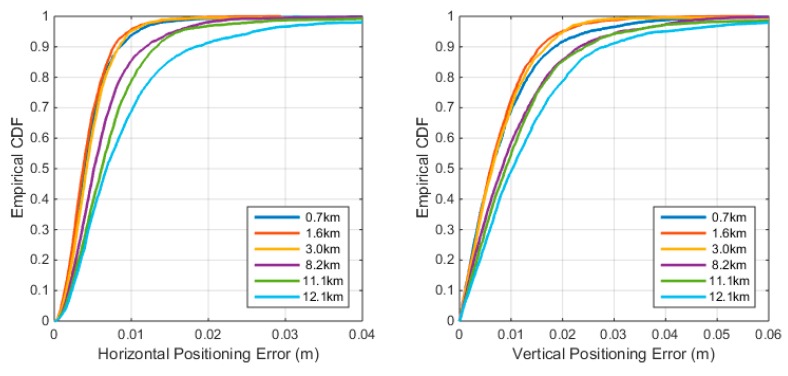
The empirical cumulative distribution function (CDF) of positioning error with difference distance to the VRS.

The true location of VRS can be extracted from type 1005 in the RTCM data stream and the true location of the monument is also precisely known. Then the positioning precision at the user side can be evaluated by comparing the positioning results and the truth. The cumulative distribution function (CDF) of the positioning error with different distances to the VRS is presented in Figure 13. The two panels show CDF of the horizontal and the vertical positioning error, respectively. The figure indicates that the positioning precision is negatively correlated to the baseline length between the VRS and user position. If the baseline length is smaller than 3 km, then the baseline length has almost no impact on the horizontal precision. A 10-km baseline reduces the 95% percentile of horizontal precision from 1 cm to 2–3 cm and reduces 95% vertical precision from 2 cm to 4 cm. For most applications, 2–3 cm horizontal precision and 4–5 cm vertical precision is acceptable. If the highest positioning accuracy required, a distance between the VRS and client no larger than 3 km is suggested.

**Figure 14:**
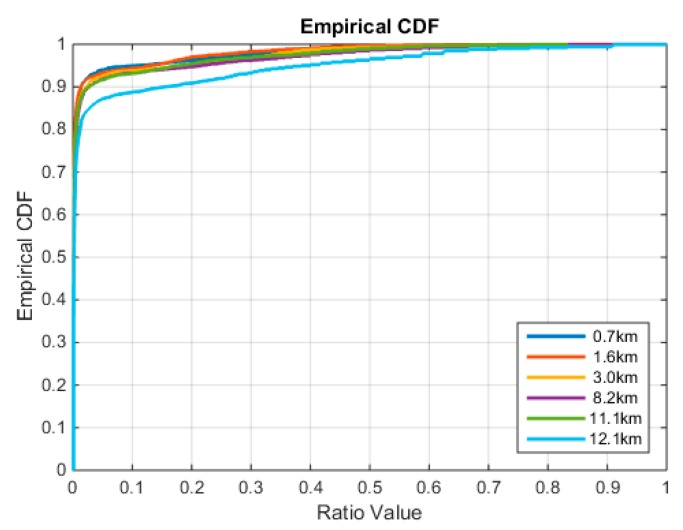
The CDF of the ratio value subjected to different distances to the VRS.

Similar to the conventional RTK system, the prerequisite for fast precise positioning for the network RTK positioning is correctly resolving the integer ambiguity parameters. Due to the remaining modeling error and the interpolation error, the precision of the float solution in network RTK is not as good as in the conventional RTK techniques. The remaining residuals in the double-differenced observations will be assimilated by the coordinate parameter and ambiguity parameter. The long baseline length between the NTRIP clients and VRS introduces additional propagation path-specified error, which affects the positioning performance by reducing the reliability of ambiguity resolution. The reliability of ambiguity resolution is often tested by various ambiguity acceptance test and the most popular test is called ratio test, which is defined as in [34]:

(7) $$ratio=\frac{\|\hat{a}-\overset{⌣}{a}{\|}_{Q_{\hat{a}\hat{a}}}^{2}}{\|\hat{a}-{\overset{⌣}{a}}_{2}{\|}_{Q_{\hat{a}\hat{a}}}^{2}}$$

where $\hat{a}$ is the float solution of ambiguity parameter, and $\overset{⌣}{a}$ and ${\overset{⌣}{a}}_{2}$ are the best and second best integer candidates, respectively. $\|\cdot {\|}_{Q_{\hat{a}\hat{a}}}^{2}$ is the squared Mahalanobis distance. The ratio test is used to discriminate the squared Mahalanobis distance between the float solution to the best and the second best integer candidate. The larger ratio means the float solution is closer to the best integer candidate and thus more likely to be the correct integer. The ratio varies between the interval [0, 1] The CDF of the ratio distribution for different baseline length case is presented in Figure 14. The figure shows that longer baseline decreases the reliability of ambiguity fixing. In contrast to the impact on positioning accuracy, the reliability of ambiguity resolution decreases more dramatically for the 12.1-km baseline, while the other baselines achieve similar ratio value. In practice, a threshold is defined to control the reliability of ambiguity resolution. If the ratio value cannot meet the threshold, the ambiguity parameter will be left as a float value, or otherwise fixed to the best integer candidate. The popular threshold for the ratio test is 1/2 or 1/3 [35]. The percentage of ambiguity fixed epochs is known as the fix rate. Table 1 lists the fix rate of baselines with different baseline lengths. In this study, the threshold is given as 1/2 and the corresponding fix rate varies from 96% to 99%, which is acceptable. Since the network RTK system transmits the real-time corrections over the Internet, the fix rate also suffers from the transmission data loss, which is listed as the outage rate. Outage rate refers to the probability of not correctly receiving the VRS corrections from the network RTK system. In this case, it is also impossible to correctly fix the ambiguity at the user side. The data outage is a pure communication problem, but it affects the fix rate evaluation.

**Figure 15:**
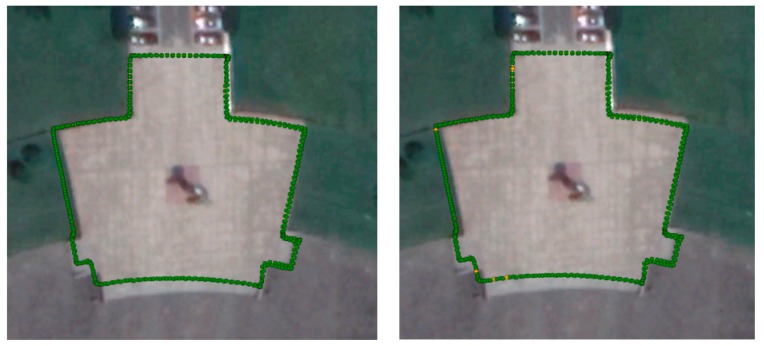
Kinematic positioning trajectory with a 5.2-km and 9.6-km baseline distance to the VRS.

In practice, most NTRIP clients are moving rather than still, so testing the positioning performance in kinematic mode is necessary. In this experiment, the individual holds a RTK surveying device and walks around a small square for two laps. The observation is recorded by the receiver and the VRS correction is collected with different distance. The kinematic positioning is processed by post-processing with the same software and configuration for comparison purposes. For convenience, the test trajectory is aligned to a small square with an irregular shape. The positioning results are compared with the satellite images from Google Earth and the results are presented in Figure 15. The two figures give the results computed from different VRS stations. The green dots indicate the fixed-ambiguity RTK solution and the yellow dots indicate the ambiguity float solution. Given a ratio of <1/2, the fixed rates for the two tests are 98.5% and 97.5%, respectively. A detailed cross-comparison of the positioning results with two different VRSs is listed in Table 2. The table reveals that the impact of different VRSs on the positioning results in terms of the standard deviation (STD) and root mean squares (RMS). The magnitude of the impact on STD and RMS is a few millimeters. The largest difference is in the upwards direction where the RMS of the two solution differences reaches 1 cm, which is negligible for most RTK applications. The results show that the distance between the user and the VRS can be safely extended to 9.6 km without significantly degrading performance. Hence, we consider that a 10-km distance between the client and VRS is safe for network RTK users and is a practical cluster radius threshold for the SOSC algorithm.

## 5. Discussion

The performance of SOSC algorithm is evaluated from both the server side and user side. At the server side, the SOSC algorithm is evaluated with respect to time consumption, cluster number, and MDTCC. Compared to the grid algorithm, the most obvious advantage of the SOSC algorithm is its elastic and automatic adaptation to the data set. Since the cluster center is predefined, its time consumption and MDTCC are independent of user distribution. Due to its insensitivity to the data set, it is almost impossible for the grid algorithm to obtain the best cluster center and cluster number for a particular data set. The advantage of the grid algorithm is its predictable time consumption and controllable cluster number. The SOSC algorithm inherits the benefits of the grid algorithm and becomes more elastic and adaptive to the dataset. The simulation results also indicate that the SOSC algorithm outperforms the grid algorithm under some particular distributions or under smaller data sets. This is because the SOSC algorithm makes changes with respect to different datasets. The results also reveal that the predefined grid approach cannot be the best choice under different scenarios.

The second aspect of the evaluation is determining the optimal cluster radius with real GNSS data. All the cluster-based approaches have reduced adverse impacts on the user positioning performance, including the grid algorithm and the SOSC algorithm. However, determination of the impact and the best cluster radius threshold did not occur. The inter-station distance in the grid algorithm is empirically determined. In this study, we find that the baseline length between the cluster center and the client should be no larger than 10 km in terms of precision and reliability. The cluster radius threshold should be no larger than 3 km to pursue the highest positioning accuracy. This conclusion is also meaningful for the grid algorithm. A 10-km cluster radius threshold is equivalent to a 10$\sqrt{2}$ km inter-station distance.

## 6. Conclusions

This paper presents a self-organizing spatial clustering (SOSC) algorithm to support large-scale network RTK systems. The current network RTK system generates virtual reference station for every online client, which represents a time-consuming procedure. The computational burden will result in a bottleneck in the system for supporting huge numbers of concurrent online users in the future. The computational burden problem can be mitigated by clustering the online users to reduce the VRS generation burden. The SOSC algorithm can cluster all online users into groups by their proximity without supervision. The test results indicate that the SOSC algorithm is comparable to the grid algorithm, and outperforms it in many cases. The grid approach guarantees the cluster number and time consumption, but it cannot ensure reasonable cluster centers since they are predefined. In contrast, the SOSC algorithm determines the cluster number and cluster center according to the data set, so it gives more reasonable clustering results. The computational efficiency, cluster number, and mean distance to cluster center (MDTCC) of the SOSC algorithm are all changed under different data distributions and data size, while the grid approach is less variable. The paper also examined the impact of clustering on the positioning performance on the user side. After clustering, the MDTCC is much larger than the single VRS for each client mode. The increased baseline has negative impact on the positioning precision and reliability. According to static and kinematic data processing, it is noted that the cluster radius can be safely extended to 10 km without significantly reducing the precision and reliability. The cluster radius threshold should no larger than 3 km for use in the highest-precision applications. From the perspective of implementation, the proposed SOSC algorithm can be implemented as middleware and seamlessly integrated into current network RTK systems without modifying existing modules. For these very large network RTK systems, the SOSC algorithm can also be deployed on a parallel computation platform. Overall, we hope the SOSC algorithm can support the large-scale network RTK system better and help to popularize the network RTK technique in future mass-market applications.

## Figures and Tables

**Table 1:** Reliability of the network RTK positioning with different distances to the VRS.

Distance to the VRS (km)	Fix Rate (Ratio <1/2)	Outage Rate
0.7	98.90%	0.40%
1.6	99.40%	0.20%
3.0	99.20%	0.00%
8.2	97.80%	0.80%
11.1	99.00%	0.00%
12.1	96.50%	0.00%

**Table 2:** The positioning difference with different baseline length between user and the VRS.

	Mean Difference (m)	STD (m)	RMS (m)
E	−0.0047	0.0047	0.0067
N	0.0059	0.0061	0.0085
U	−0.0062	0.0079	0.0100
